# Trimetallic ferrite functionalized by guaninium tartrate ionic liquid (Co_0.2_Zn_0.6_Cu_0.2_Fe_2_O_4_-SiO_2_@[GuaH]^+^[Tar]^2‒^[GuaH]^+^) as a novel inorganic-bioorganic nanostructure to promote aqua-mediated synthesis of polyhydroxy-substituted pyridine-dipyrimidine fused heterocycles

**DOI:** 10.1016/j.heliyon.2025.e42462

**Published:** 2025-02-04

**Authors:** Zahra Khademi, Kobra Nikoofar, Mansoureh Zahedi-Tabrizi

**Affiliations:** a Department of Organic Chemistry, Faculty of Chemistry, Alzahra University, Tehran, Iran; b Department of Physical Chemistry & Nanochemistry, Faculty of Chemistry, Alzahra University, Tehran, Iran

## Abstract

In this work, a Cobalt-Zinc-Copper ferrite (Co_0.2_Zn_0.6_Cu_0.2_Fe_2_O_4_, CZCF) was synthesized and functionalized with silica and guaninium tartrate ionic liquid (Co_0.2_Zn_0.6_Cu_0.2_Fe_2_O_4_-SiO_2_@[GuaH]^+^[Tar]^2‒^[GuaH]^+^). The novel bio-nanostructure was characterized by various techniques such as fourier transform infrared spectroscopy (FT-IR), energy dispersive X-ray analysis (EDAX), EDAX mapping, field emission scanning electron microscopy (FESEM), X-ray fluorescence (XRF), X-ray diffraction (XRD), thermogravimetric/differential thermal gravimetric analysis (TGA/DTG), vibrating sample magnetometry (VSM), high resolution transmission electron microscopy (HRTEM), and zeta potential analysis. The synthesized bio-nanocomposite exhibited high catalytic activity for the aqua-mediated synthesis of polyhydroxy-substituted pyridine-dipyrimidine fused heterocycles through the one-pot pseudo four-component reaction of carbohydrates (sugars), barbituric acid, and amines under refluxing conditions. The recyclability and reusability of the bio-nanocatalyst were successfully investigated for up to three runs. Moreover, the features of the recovered Co_0.2_Zn_0.6_Cu_0.2_Fe_2_O_4_-SiO_2_@[GuaH]^+^[Tar]^2‒^[GuaH]^+^ were examined via the EDAX analysis and FESEM images. In the theoretical section, the interaction sites between *L*-tartaric acid and guanine in an aqueous medium were investigated at the B3LYP/6–311++G(d,p) computational level. Additionally, the formation of more stable configurations of dimers and trimers in IL was studied from a thermodynamic point of view.

## Graphical abstract

**Figure:**
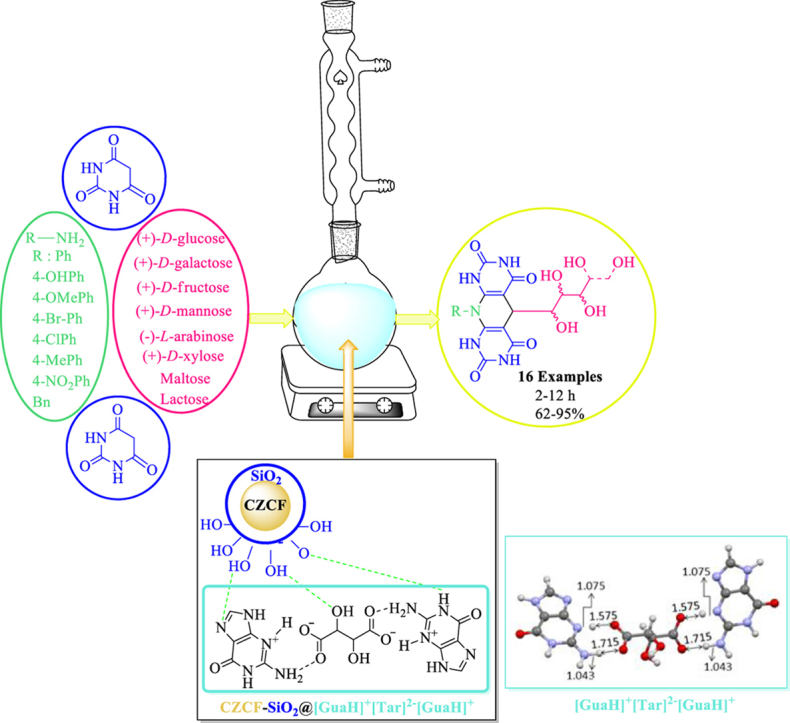

## 1 Introduction

Magnetized nano spinel ferrites with the general chemical formula of MFe_2_O_4_ (M = Ni, Zn, Mg, Co, Mn, Ta, etc.) possess diverse and distinctive properties such as thermal and chemical stability, high magnetization, anisotropy, optical, and electrical characteristics, and great dielectric constant. Various kinds of nano ferrites are comprehensively investigated due to their widespread significant applications in various fields of science and technology such as targeted drug delivery and hyperthermia [1], electrochemical sensors [2], and wastewater treatment [3].

Nano spinel ferrites also demonstrated crucial catalytic applications. They were reported as reusable green catalysts to promote multi-component reactions [4]. Their catalytic potential in the environment and energy fields has also been investigated previously [5]. They can be utilized as photocatalysts and adsorbents for the degradation of organic pollutants [6]. They are also applicable as novel catalysts for visible light-assisted dye degradation [7].

The MFe_2_O_4_ could be prepared through various techniques, such as electrochemical, microemulsion, sol-gel, hydrothermal, solid-state, sono-chemical, precipitation, co-precipitation, auto-combustion, and template methods [[8], [9], [10]].

Recently, diverse ferrite-based nanohybrids/composites have gained significant attention in various fields of applications [11]. Some examples are: the CoFe_2_O_4_/Ag-fMWCNT hybrid nanocomposite to degrade synthetic organic dyes via peroxymonosulfate activation [12], copper ferrite nanosphere composites mixed with carbon black (CFNs/vulcan) to boost the oxygen reduction reaction [13], CoFe_2_O_4_@SiO_2_-CPTES-melamine nanoparticles to catalyze the synthesis of dihydropyrano[2,3-*c*]pyrazoles [14], facilitative reduction and reductive acetylation of various nitroarenes as well as the acetylation of aromatic amines by the rigid SbF_x_ species on copper or nickel ferrite (CuFe_2_O_4_@SbF_x_ and NiFe_2_O_4_@SbF_x_) [15], hydration of nitriles and oxidative decarboxylation of phenylacetic acids by the CoFe_2_O_4_@SiO_2_ nanocomposite [16], oxidation of styrene by the mesoporous titanosilicate-silica-coated cobalt ferrite core-shell (Ti-SiO_2_@CF) [17], and solvent-free Knoevenagel condensation by nitrogen-enriched biguanidine-functionalized cobalt ferrite nanoparticles (CoFe_2_O_4_@SiO_2_@NH_2_@BG) [18].

Bio-based ionic liquids (Bio-ILs) are a class of ILs that consist of natural and/or bioderived compounds as part of their structure. In fact, the highlighted strategies for their synthesis involve utilizing molecules from bio-renewable sources as their anionic and/or cationic counterparts. Some species of bio-ILs (which are actually task-specific) are: amino acid-based ionic liquids (AAILs) [19], carbohydrate-derived ILs [20], and choline-based ILs [21]. The bio-ILs possess special properties, such as low toxicity, expanded solvation nature, high ionic conductivity, renewability, multi-functionality, chirality, relatively low viscosity, eco-/bio-friendliness, and biodegradability. They play a role in various fields, such as lubricants (with excellent anti-wear and friction reduction properties) [22], pharmaceutics and medicine (as potent antibacterial and anticancer compounds) [23,24], and catalysis/reaction media [25].

Guanine (2-amino-1,9-dihydro-6*H*-purin-6-one, G, Gua) is one of the four major nucleobases found in DNA. Guanine is a derivative of the purine base that includes a fused pyrimidine-imidazole ring system along with conjugated double bonds [26]. In 2020, a novel bio-based core-shell organic-inorganic nanohybrid created by embedding aspartic acid-guanine ionic liquid on the hydroxylated nano silica surface (nano [(Asp-Gua) IL@PEG-SiO_2_]) promoted the synthesis of bis(2,3-dihydroquinazolin-4(1*H*)-one) derivatives and tricarboxamides [27].

Carbohydrates are eco-friendly precursors/organics due to their unique features such as accessibility, optical activity, and high-water solubility. They possess interesting applications as organocatalysts [28,29], HIV vaccine candidates [30], in the synthesis of macromolecular biomaterials [31], in mitigating virus-induced complications (such as bacterial infection, cardiovascular disorders, oxidative stress, and metabolic disorders) [32], chronic wound treatment [33], breast cancer therapy [34], and drug delivery [35,36]. Additionally, carbohydrates are versatile substances for the synthesis of diverse heterocycles and/or bioactive compounds via multi-component reactions [37,38].

**Scheme 1:**
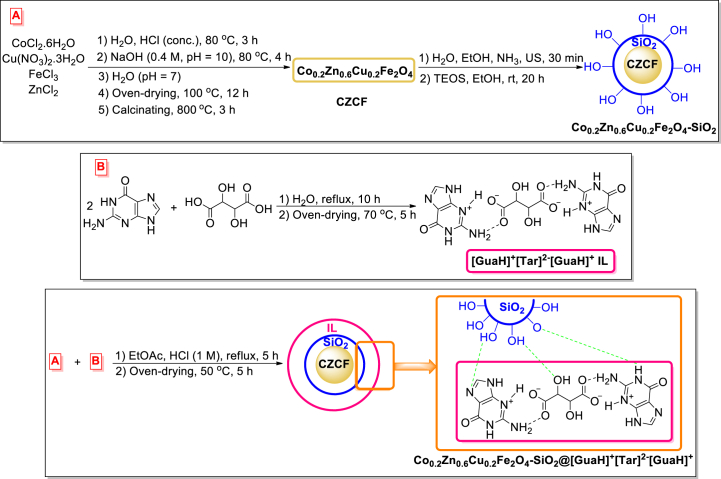
Schematic synthetic procedure steps of Co_0.2_Zn_0.6_Cu_0.2_Fe_2_O_4_-SiO_2_@[GuaH]^+^[Tar]^2‒^[GuaH]^+^ bio-nanocomposite.

**Scheme 2:**
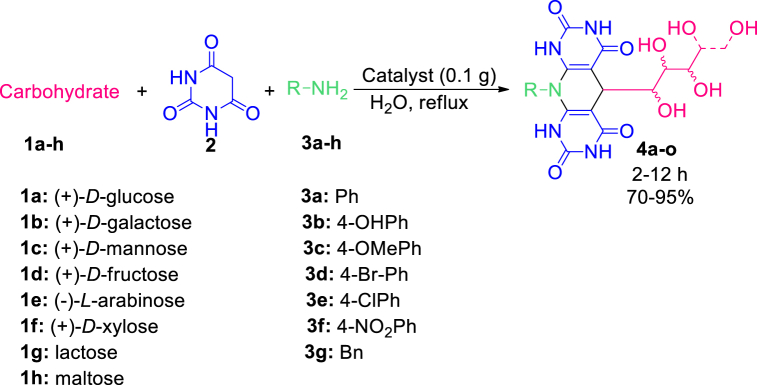
Synthesis of polyhydroxy-substituted pyridine-dipyrimidine fused heterocycles **4a-**-o in the presence of C_2_ZCF-SiO_2_@[GuaH]^+^[Tar]^2‒^[GuaH]^+^ bio-nanocomposite.

In this research, a novel magnetized inorganic-bioorganic nanohybrid was synthesized that consists of cobalt-zinc-copper ferrite, silica, and guaninium tartrate ionic liquid, (Co_0.2_Zn_0.6_Cu_0.2_Fe_2_O_4_-SiO_2_@[GuaH]^+^[Tar]^2‒^[GuaH]^+^, CZCF-SiO_2_@[GuaH]^+^[Tar]^2‒^[GuaH]^+^) (Scheme 1). The synthesized bio-nanocomposite was investigated to promote the green aqua-mediated synthesis of polyhydroxy-substituted pyridine-dipyrimidine fused heterocyclic compounds under reflux conditions (Scheme 2).

## 2 Results and discussion

### 2.1 Characterization of the catalyst

The schematic synthetic procedure of the multi-layered Co_0.2_Zn_0.6_Cu_0.2_Fe_2_O_4_-SiO_2_@[GuaH]^+^[Tar]^2‒^[GuaH]^+^ bio-nanocomposite is illustrated in Scheme 1. To clarify the structure of the novel IL, DFT calculations have been done as follows.

**Fig. 1:**
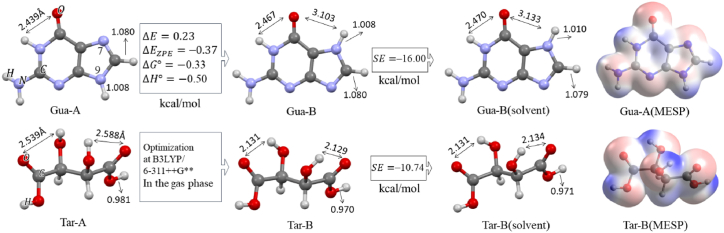
The optimized structures of Gua-A and Gua-B, the optimized structure of Gua-B in the aqueous phase, the crystal structure of L-tartaric acid (Tar-A)^42^ and its optimized structures (Tar-B) in both phases, and MESP of Gua-A and Tar-B in the gas phase calculated at B3LYP/6–311++G(d,p) level.

The fully optimized structure of the guanine (COD Number: 9011114) at B3LYP/6–311++G (d,p) computational level (Gua-A) in the gas phase is shown in Fig. 1. The optimization process of Gua-A was based on the X-ray crystallographic data of guanine monohydrate [39]. The difference between the total electronic energy of Gua-A and Gua-B (ΔE) tautomers and their zero-point corrected energy values is shown in Fig. 1. The calculated standard Gibbs free energy change of this tautomeric exchange in the gas phase indicates that both structures can exist in the gas phase with a small energy difference. Additional research on the tautomeric forms of Gua [40,41] revealed that Gua-B (Keto N7H) was slightly more stable than the Gua-A (Keto N9H) tautomer. Fig. 1 represents the optimized structure of Gua-B in the water solvent along with its solvation energy. The Gibbs free energy change comparison indicates that Gua-B is more stable than Gua-A (0.33 kcal/mol) in the gas phase whereas Gua-A is more stable than Gua-B (0.61 kcal/mol) in the solvent.

The crystal structure of *L*-tartaric acid, Tar-A (COD Number: 7241734), is shown in Fig. 1. The optimization process of tartaric acid was initiated from the Tar-A [42] structure. After optimization in the gas phase, the molecular conformation changed to the Tar-B conformer, which was in agreement with the most abundant conformer observed for this compound [43]. The solvation energy of the Tar-B conformer was obtained to be −10.74 kcal/mol. The molecular electrostatic potential (MESP) maps of Gua-A and Tar-B in Fig. 1 show a three-dimensional perspective of the abundance of electrons in these molecules. In this figure, the red regions indicate low electrostatic potential with the accumulation of electrons, while the blue regions show high electrostatic potential with low electron density. These maps could predict the interaction sites of nucleophilic and electrophilic attacks.

**Fig. 2:**
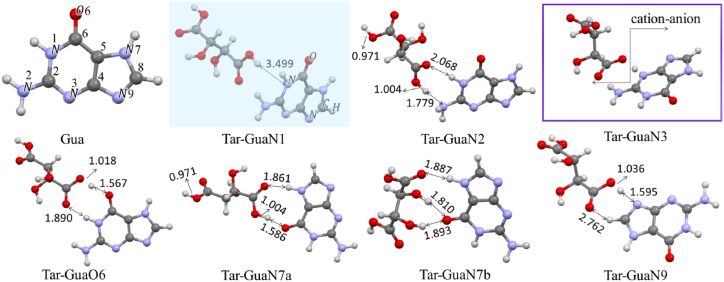
The numbering system of the optimized structures of Gua and more stable Tar-Gua complexes.

**Fig. 3:**
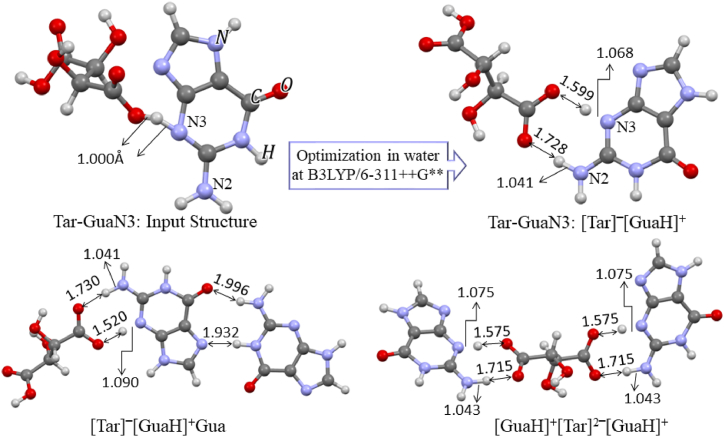
The optimized structures of the most stable ILs in an aqueous phase.

In the next section of the theoretical analysis, the different interaction sites of Gua-B and Tar-B were inspected by considering the MESP maps of the two molecules and the possibility of IHB formation. The optimized structure of Gua-B in the solvent along with its numbering system (Gua), as well as more stable configurations are shown in Fig. 2. In their nomenclature, Gua's nitrogen label signifies the selected interaction site before starting the optimization procedures. In the initial structure of these complexes, we focused on the neutral molecules in which the hydrogen atom of the carboxyl group of Tar-B was exactly substituted between two proton acceptors (similar to Tar-Gua3 in Fig. 3). All optimization processes were carried out in the aqueous phase at the same level of theory and frequency calculations were performed to confirm the global minimum in potential energy surface. The optimized structures of all complexes together with IHB lengths are shown in Fig. 2. Table 1 lists the calculated interaction energies according to Eq. (1), along with the zero-point corrected interaction energies of all complexes.

**Table 1:** The interaction energies ($E_{\mathrm{int}}$), zero-point corrected interaction energies ($E_{\mathrm{int}}$, ZP Corr.), and thermodynamic functions of binding at 298.15K and 1.0 atm. All parameters in kcal/mol; ΔS° in cal/mol.K.

Conf.	$E_{\mathrm{int}}$	$E_{\mathrm{int}}$ ZP Corr.	ΔG°	ΔH°	ΔS°
Tar-GuaN2	−6.37	−4.26	6.49	−3.89	−34.84
Tar-GuaN3	−13.07	−11.50	−0.17	−11.27	−37.23
Tar-GuaO6	−11.59	−10.27	0.72	−9.77	−35.18
Tar-GuaN7a	−12.02	−10.64	0.42	−10.14	−35.42
Tar-GuaN7b	−8.98	−6.52	5.78	−6.39	−40.82
Tar-GuaN9	−10.61	−9.71	0.36	−9.04	−31.56
ILs					
[Tar]‾[GuaH]^+^	−19.43	−19.31	−8.38	−18.91	−35.35
[Tar]‾[GuaH]^+^Gua	−28.64	−27.81	−5.65	−26.96	−71.50
[GuaH]^+^[Tar]^2^‾[GuaH]^+^	−42.01	−42.21	−18.55	−42.02	−78.71
[GuaH]^+^[Tar]‾[GuaH]^+^	−31.99	−30.99	−10.51	−29.93	−65.15

Comparing the interaction energies and the binding Gibbs free energy changes of six configurations in Fig. 2 shows that Tar-GuaN3 is the most stable complex. The interaction sites of O6, N7a, and N9 with the binding standard Gibbs free energy changes lower than 0.72 kcal/mol could be thermodynamically close to equilibrium conditions. All binding processes are exothermic and, their standard entropy changes are negative, likely due to the formation of a complex from two separate molecules. Our previous results for calculating the interaction energies and binding thermodynamic parameters showed that although their exact values are related to the computational level [44], the increasing trend in similar interactions remains almost identical.

Fig. 2 shows that the interaction of the Tar molecule with the N1 site of Gua (Tar-GuaN1) is not possible and during optimization, the O…N distance was achieved more than 3.50 Å. The calculated O…H-N and O…H-O bond lengths and bond angles lie within the normal range of hydrogen bond lengths [45,46]. These results indicate that all interactions in Fig. 2 could be IHBs with different strengths except for Tar-GuaN3, for which a detailed analysis of its interaction sites is presented in Fig. 3. This figure shows that in the optimized structure of Tar-GuaN3 in water, the Tar's proton was completely transferred to the N3 atom of Gua. Therefore, to calculate the interaction energies and thermodynamic parameters, we have considered Eq. (2). These parameters are collected in the ILs of Table 1. For cation-anion interactions we have considered [GuaN3H]^+^ (here after [GuaH]^+^) cation, [Tar]‾, and [Tar]^2^‾ anions. The most stable trimer structures of ILs are shown in Fig. 3. In [Tar]‾[GuaH]^+^Gua trimer we have considered the Gua-A structure due to the availability of crystallographic data of its IHBs and the proper orientation of two Gua-A toward each other. Table 1 reveals that the [Tar]^2^‾ anion can act as a bridge between two [GuaH]^+^ cations, with a zero-point corrected interaction energy of 42.21 kcal/mol. The binding standard Gibbs free energy of this trimer (−18.55 kcal/mol) in the aqueous phase confirms a very stable complex in this IL.

**Fig. 4:**
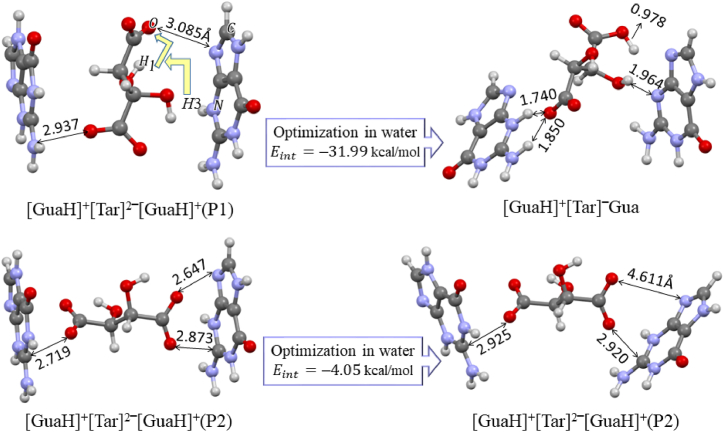
P1 and P2 configurations [GuaH]^+^[Tar]^2^‾[GuaH]^+^ before and after optimization in an aqueous phase.

In the last section of the theoretical studies, two configurations of ILs that [Tar]^2^‾ anion is placed between two parallel [GuaH]^+^ cations were evaluated. These configurations (P1 and P2) before and after optimization in aqueous medium along with their interaction energies are shown in Fig. 4. The parallel configuration [GuaH]^+^[Tar]^2^‾[GuaH]^+^ (P2) has been excluded from ILs of Table 1 due to its low interaction energy. In the first optimization step in the aqueous medium for the [GuaH]^+^[Tar]^2^‾[GuaH]^+^ (P1) configuration, the H1 atom of [Tar]^2^‾ was attached to the oxygen atom of [Tar]^2^‾, and simultaneously, the H3 proton of the [GuaH]^+^ cation was replaced in the H1 vacancy (Fig. 4). Therefore, we have considered [GuaH]^+^[Tar]‾Gua label for the optimized structure of this configuration.

The optimized structure of [GuaH]^+^[Tar]‾Gua in Fig. 4 shows that the directionality of Gua and [GuaH]^+^ are rearranged to provide the best sites for cation-anion interaction and IHB formation. The calculated thermodynamic parameters of this configuration are also displayed in the last row of Table 1. The obtained parameters indicate that the formation of this configuration is also thermodynamically favorable, with the binding standard Gibbs free energy of 8.04 kcal/mol lower than the [GuaH]^+^[Tar]^2^‾[GuaH]^+^ configuration. It should be noted that the study of interactions in ILs is very complicated due to solvent effects, interactions with water molecules, diversity of configurations, the existence of neutral molecules, different numbers of anions and cations at different pH levels of the solution, concentration effects, etc. In this theoretical study, we have considered a comprehensive view of the most stable configurations in a polarizable continuum model of solvent.

**Fig. 5:**
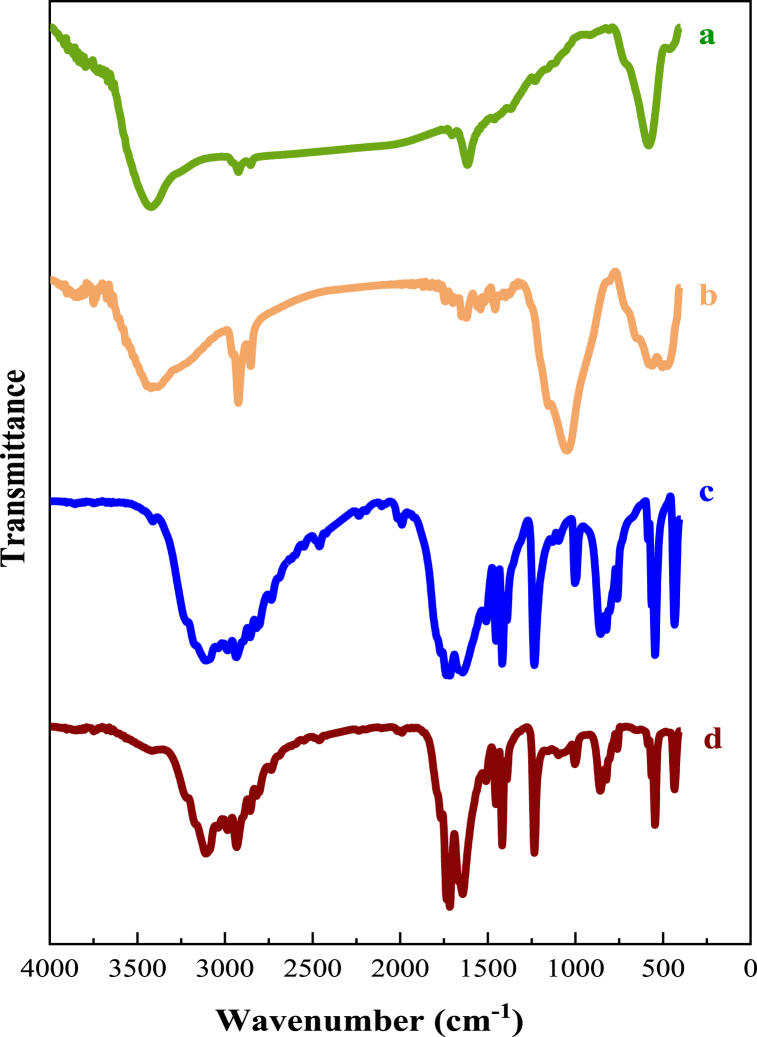
FT-IR spectra of Co_0.2_Zn_0.6_Cu_0.2_Fe_2_O_4_ (a), Co_0.2_Zn_0.6_Cu_0.2_Fe_2_O_4_-SiO_2_ (b), IL (c), and Co_0.2_Zn_0.6_Cu_0.2_Fe_2_O_4_-SiO_2_@[GuaH]^+^[Tar]^2‒^[GuaH]^+^ (d).

The FT-IR spectra of Co_0.2_Zn_0.6_Cu_0.2_Fe_2_O_4_ (a), Co_0.2_Zn_0.6_Cu_0.2_Fe_2_O_4_-SiO_2_ (b), IL (c), and Co_0.2_Zn_0.6_Cu_0.2_Fe_2_O_4_-SiO_2_@[GuaH]^+^[Tar]^2‒^[GuaH]^+^ bio-nanocomposite (d) are shown in Fig. 5. According to Fig. 5a, the broad bands at 3419 cm^−1^ and 1616 cm^−1^ are attributed to the stretching and bending vibrations of the OH group (related to the surface absorbed water), respectively. The peaks at 581 and 463 cm^−1^ are ascribed to the tetrahedral and octahedral vibrations of Fe-O bands, respectively [47]. In Fig. 5b, the strong absorption band at 1048 cm^−1^ (Si–O–Si stretching vibration), the very weak peak at 790 cm^−1^ (Si–O–Si bending vibration), and the relatively strong peak at 466 cm^−1^ (Si–O–Si rocking vibration), affirm the coating of silica on the Co_0.2_Zn_0.6_Cu_0.2_Fe_2_O_4_ core [48]. According to Fig. 5c, the broad peak in the range of 3110 cm^−1^ to 2460 cm^−1^ (stretching vibration of NH^+^ and NH), 1508 cm^−1^ and 856 cm^−1^ (bending vibrations of NH), 1724 cm^−1^ and 1417 cm^−1^ (the asymmetric and symmetric stretching bands of COO ^-^), 2735 cm^−1^ (the aliphatic C-C stretching vibration), 1233 cm^−1^ (the C-N stretching vibration), and 1645 cm^−1^ (the C=N stretching vibration), affirm the successful synthesis of the IL. The FT-IR spectra of the final bio-nanocomposite (Fig. 5d) include all the peaks of the previous layers, with some overlapping.

**Fig. 6:**
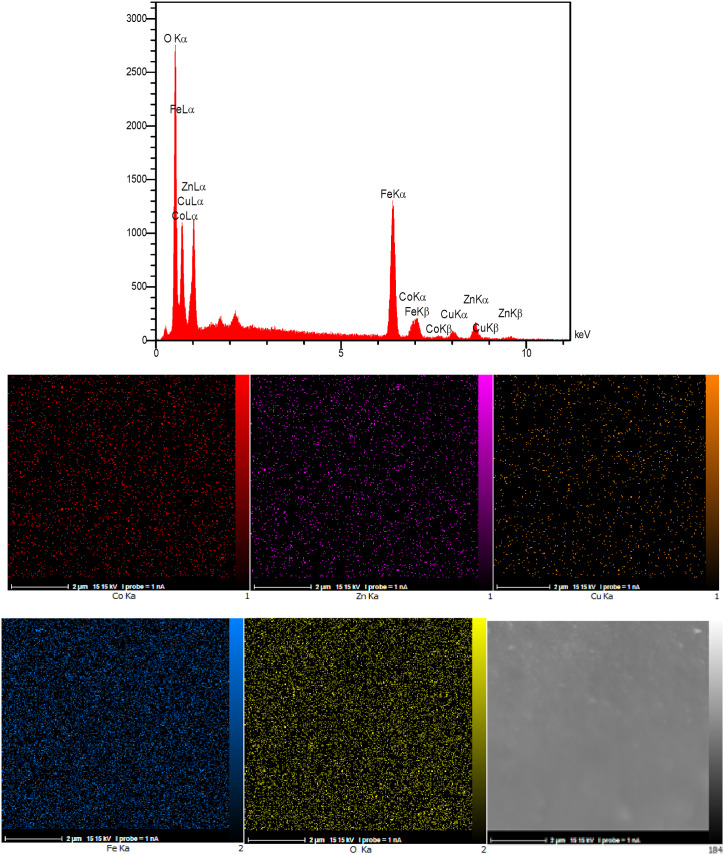
EDAX analysis and elemental mapping of Co_0.2_Zn_0.6_Cu_0.2_Fe_2_O_4_.

The EDAX analysis and the elemental mapping of the Co_0.2_Zn_0.6_Cu_0.2_Fe_2_O_4_ core are illustrated in Fig. 6. The presence of cobalt (4.38 wt%), zinc (13.57 wt%), copper (4.42 wt%), iron (41.18 wt%), and oxygen (36.45 wt%) without any other impurities revealed the core preparation. In addition, the elemental mapping patterns reveal the good dispersion of the elements throughout the Co-Zn-Cu ferrite.

**Fig. 7:**
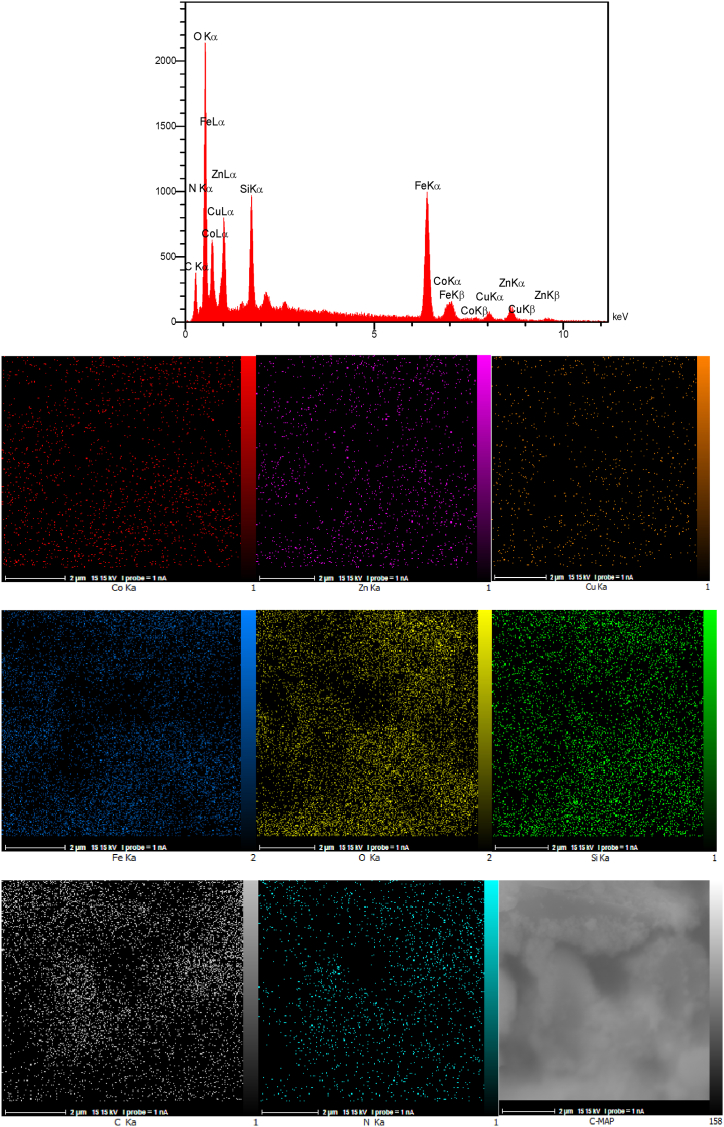
EDAX analysis and elemental mapping of Co_0.2_Zn_0.6_Cu_0.2_Fe_2_O_4_-SiO_2_@[GuaH]^+^[Tar]^2‒^[GuaH]^+^.

The EDAX analysis of Co_0.2_Zn_0.6_Cu_0.2_Fe_2_O_4_-SiO_2_@[GuaH]^+^[Tar]^2‒^[GuaH]^+^ in Fig. 7 confirms the presence of cobalt (2.95 wt%), zinc (8.05 wt%), copper (2.87 wt%), iron (25.36 wt%), oxygen (35.60 wt%), silicon (3.74 wt%), carbon (16.45 wt%), and nitrogen (4.97 wt%). These results validate the successful fabrication of the multi-layered bio-nanocomposite. Based on the mapping patterns, all the elements are uniformly distributed within the bio-nanostructure.

**Fig. 8:**
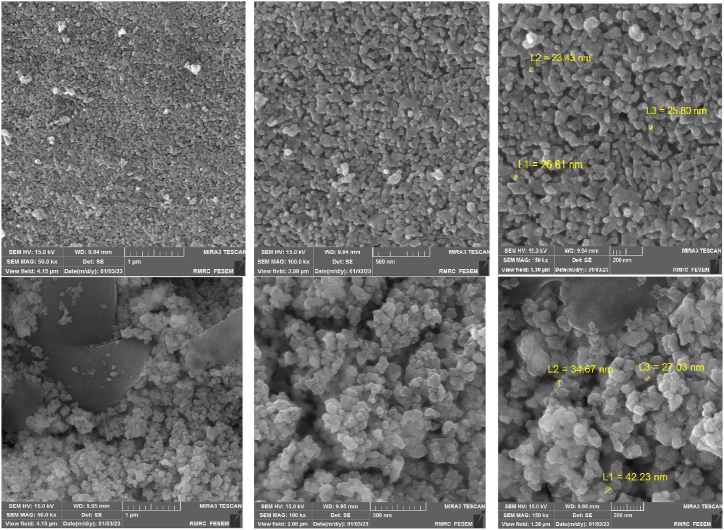
FESEM images of Co_0.2_Zn_0.6_Cu_0.2_Fe_2_O_4_ core (top) and Co_0.2_Zn_0.6_Cu_0.2_Fe_2_O_4_-SiO_2_@[GuaH]^+^[Tar]^2‒^.

The FESEM images (at 1 μm, 500 nm, and 200 nm magnification scales) of the Co_0.2_Zn_0.6_Cu_0.2_Fe_2_O_4_ core (top) and the final magnetized bio-nanocatalyst Co_0.2_Zn_0.6_Cu_0.2_Fe_2_O_4_-SiO_2_@[GuaH]^+^[Tar]^2‒^[GuaH]^+^ (bottom) are represented in Fig. 8. Based on the top images, the core exhibited almost uniform nano-sized particles with an average size of 20–25 nm. The final bio-nanocomposite consisted of semi-uniform and pseudo-spherical nano-sized particles with an average diameter of 30–40 nm. The increased particle size indicates embedding of the silica and bio-IL layers on the ferrite core.

**Fig. 9:**
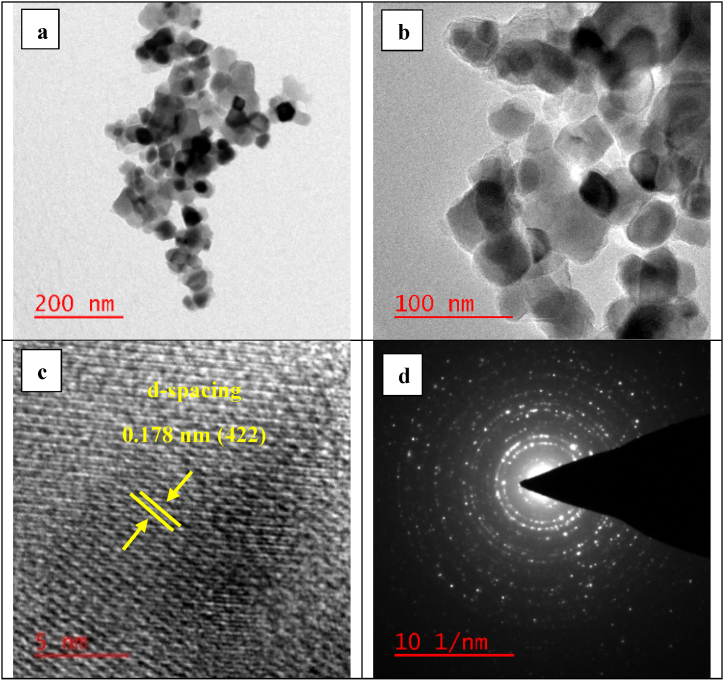
HRTEM images (a)/(b), HRTEM of interplanar distances (c), and SAED micrograph (d) of the CZCF-SiO_2_@[GuaH]^+^[Tar]^2‒^[GuaH]^+^.

The HRTEM images of the bio-nanocomposite are shown in Fig. 9a–d. The micrographs exhibited in Fig. 9a and b (at 200 nm and 100 nm magnification) demonstrate that the morphology of the particles is mostly spherical. Also, the structural information has been obtained from the lattice fringes (Fig. 9c) and the selected area electron diffraction (SAED) pattern (Fig. 9d). According to Fig. 9c, it is evident that the lattice fringes are related to a class of atomic planes within the particle, indicating that the particle is a single crystalline without any deficiencies. The distance between the two adjoining planes is measured at approximately 0.178 nm, which is associated to the (422) plane of the mixed spinel phase. The SAED pattern shown in Fig. 9d demonstrates the polycrystalline nature of the bio-nanocomposite.

The XRF analysis of the Co_0.2_Zn_0.6_Cu_0.2_Fe_2_O_4_ core and Co_0.2_Zn_0.6_Cu_0.2_Fe_2_O_4_-SiO_2_ are presented in Table 2 (entries 1 and 2, respectively). According to entry 1, the core structure revealed the presence of Fe_2_O_3_ (69.123 %), Zn (17.911 %), Cu (5.558 %), and Co (5.237. Entry 2 in Table 2 confirmed that Co_0.2_Zn_0.6_Cu_0.2_Fe_2_O_4_-SiO_2_ was composed of Fe_2_O_3_ (62.296 %), SiO_2_ (10.537 %), Zn (16.39 %), Cu (5.129 %), and Co (4.917 %). Based on the resulting data, the presence of SiO_2_ attested to the successful synthesis of Co_0.2_Zn_0.6_Cu_0.2_Fe_2_O_4_-SiO_2_ through the immobilization process on the initial Co_0.2_Zn_0.6_Cu_0.2_Fe_2_O_4_ core.

**Table 2:** XRF analysis of Co_0.2_Zn_0.6_Cu_0.2_Fe_2_O_4_ core and Co_0.2_Zn_0.6_Cu_0.2_Fe_2_O_4_-SiO_2_.

Entry	Fe_2_O_3_ (%)	SiO_2_ (%)	Zn (%)	Cu (%)	Co (%)
1	69.123	–	17.911	5.558	5.237
2	62.296	10.537	16.39	5.129	4.917

**Fig. 10:**
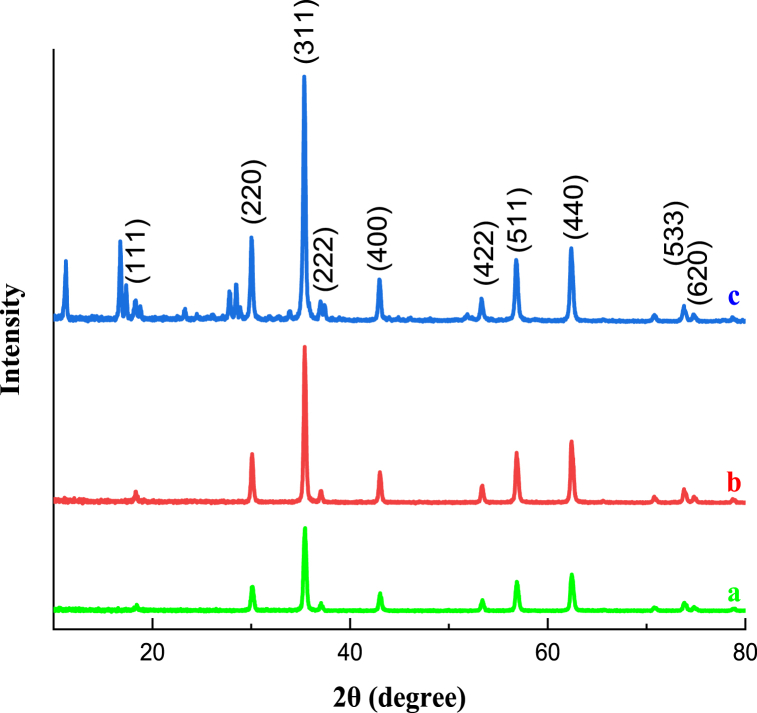
XRD pattern of Co_0.2_Zn_0.6_Cu_0.2_Fe_2_O_4_ (a), Co_0.2_Zn_0.6_Cu_0.2_Fe_2_O_4_-SiO_2_ (b), and Co_0.2_Zn_0.6_Cu_0.2_Fe_2_O_4_-SiO_2_@[GuaH]^+^[Tar]^2‒^[GuaH]^+^ (c).

The XRD diagrams of Co_0.2_Zn_0.6_Cu_0.2_Fe_2_O_4_ (a), Co_0.2_Zn_0.6_Cu_0.2_Fe_2_O_4_-SiO_2_ (b), and Co_0.2_Zn_0.6_Cu_0.2_Fe_2_O_4_-SiO_2_@[GuaH]^+^[Tar]^2‒^[GuaH]^+^ (c) are presented in Fig. 10. As shown in Fig. 10a, the diffraction angles at the mentioned plates 18.3° (111), 30° (220), 35.4° (311), 37° (222), 43° (400), and 53.34° (422), 56.84° (511), and 62.39° (440) affirm the formation of the mixed spinel Co-Zn-Cu ferrite phase [49]. In Fig. 10b, the slight broad peak at 18–30 confirms the presence of amorphous silica. The XRD of the bio-nanocatalyst (Fig. 10c), consists of invariant peaks of core and silica along with the appearance of some peaks related to guanine at 17.41°, 23.25°, 27.43°, and 28.72° [50] and *L*-tartaric acid at 11.07°, 18.81°, 16.79°, 33.98°, and 33.35° [51]. The results exhibit the successful synthesis of the final bio-nanocomposite without structural changes.

**Fig. 11:**
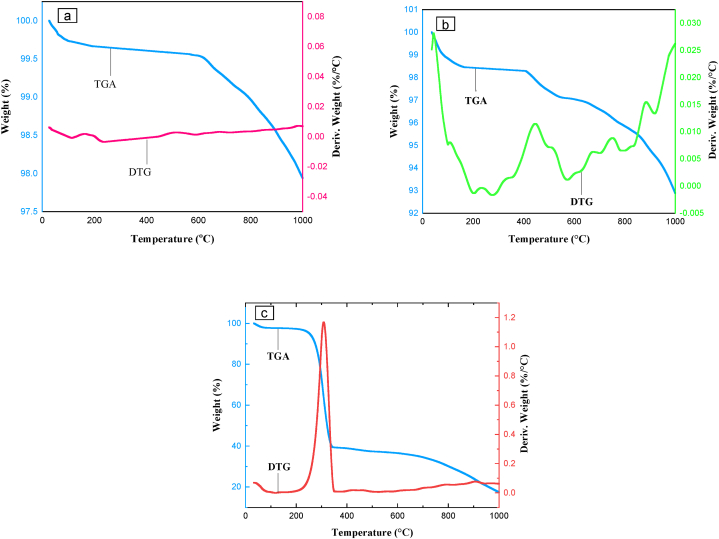
TGA/DTG curves of Co_0.2_Zn_0.6_Cu_0.2_Fe_2_O_4_ MNPs (a), Co_0.2_Zn_0.6_Cu_0.2_Fe_2_O_4_-SiO_2_ (b), and Co_0.2_Zn_0.6_Cu_0.2_Fe_2_O_4_-SiO_2_@[GuaH]^+^[Tar]^2‒^[GuaH]^+^ (c).

As could be seen in Fig. 11, the thermal behavior of Co_0.2_Zn_0.6_Cu_0.2_Fe_2_O_4_ (a), Co_0.2_Zn_0.6_Cu_0.2_Fe_2_O_4_-SiO_2_ (b), and Co_0.2_Zn_0.6_Cu_0.2_Fe_2_O_4_-SiO_2_@[GuaH]^+^[Tar]^2‒^[GuaH]^+^ (c) was examined via TGA/DTG diagrams up to 1000 °C. According to Fig. 11a, the initial weight loss occurred at 164.27 °C (0.31 %). The second weight loss was observed at 513.96 °C (0.42 %). Heating the Co_0.2_Zn_0.6_Cu_0.2_Fe_2_O_4_ core up to 1000 °C led to a total weight loss of 2.090 %. The TGA/DTG curves of Co_0.2_Zn_0.6_Cu_0.2_Fe_2_O_4_-SiO_2_ in Fig. 11b exhibited four weight loss stages at 43.49 °C (0.16 %), 446.03 °C (2.1 %), 751.12 °C (3. 8 %), and 885.18 °C (4.97 %). Heating up to 1000 °C, resulted in a total weight loss of 7.23 %. Based on Fig. 11c, the mass loss of about 36.8 % at 309 °C, is related to the decomposition of the bioorganic outer layer of bio-nanocomposite ([GuaH]^+^[Tar]^2‒^[GuaH]^+^). Heating up to 1000 °C yielded a total weight loss of 82.87 % which corresponds to the complete decomposition of bio-nanocomposite.

**Fig. 12:**
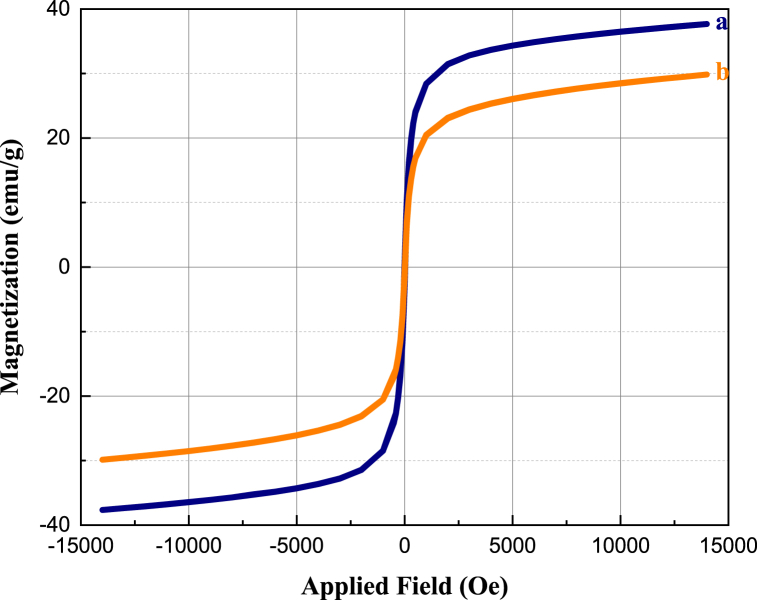
Magnetization curves of Co_0.2_Zn_0.6_Cu_0.2_Fe_2_O_4_ (a), and Co_0.2_Zn_0.6_Cu_0.2_Fe_2_O_4_-SiO_2_@[GuaH]^+^[Tar]^2‒^[GuaH]^+^ (b).

As shown in Fig. 12, the magnetic characteristics of the Co_0.2_Zn_0.6_Cu_0.2_Fe_2_O_4_ core and the final bio-nanocomposite are investigated through VSM analysis. On the basis of the resulting data, the curves correspond to Co_0.2_Zn_0.6_Cu_0.2_Fe_2_O_4_ (a), and Co_0.2_Zn_0.6_Cu_0.2_Fe_2_O_4_-SiO_2_@[GuaH]^+^[Tar]^2‒^[GuaH]^+^ (b) displaying saturation magnetization values of 37.688 emu g^−1^ and 29.850 emu g^−1^, respectively. Stabilizing the silica and IL layers on the core reduced its magnetization due to the coating of the magnetic core with diamagnetic layers.

**Fig. 13:**
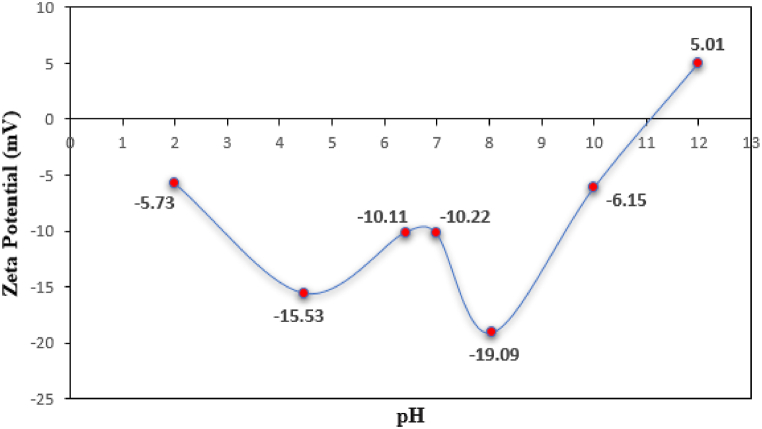
zeta potential vs. pH values of Co_0.2_Zn_0.6_Cu_0.2_Fe_2_O_4_-SiO_2_@[GuaH]^+^[Tar]^2‒^[GuaH]^+^.

The pH of the Co_0.2_Zn_0.6_Cu_0.2_Fe_2_O_4_-SiO_2_@[GuaH]^+^[Tar]^2‒^[GuaH]^+^ bio-nanocomposite has been studied via zeta potential analysis. According to Fig. 13, which showed the zeta potential changes versus pH, the isoelectric point of the bio-nanocomposite is at pH = 11.

### 2.2 Investigation the catalytic activity of CZCF-SiO_2_@[GuaH]^+^[Tar]^2‒^[GuaH]^+^ for the synthesis of polyhydroxy-substituted pyridine-dipyrimidine fused heterocycles

To optimize the conditions, the one-pot three-component condensation reaction of lactose (**1g**, 1 mmol), barbituric acid (**2**, 2 mmol), and aniline (**3a**, 1 mmol) was selected as the model reaction. Based on the data in Table 3, different parameters were investigated to access **4n**. According to the data, the presence of the solvent and temperature are efficient for the reaction progress. Hence, performing the reaction under aqua-mediated reflux conditions is the best choice (entry 5). The amount of the catalyst was another tested parameter (entries 8–10), where the presence of 0.1 g of bio-nanocomposite was sufficient (entry 5). The reported times are related to the maximum reaction progress and extending the reaction time did not affect further reaction promotion.

**Table 3:** Optimization of the reaction conditions for the preparation of **4n**.


Entry	Bio-nanocomposite (g)/solvent (3 mL)/temperature (^o^C)	Time (h)	Yield (%)
1	0.1/-/rt	8	–
2	0.1/-/80	8	40
3	0.1/-/100	2	40
4	0.1/H_2_O/rt	6	30
5	0.1/H_2_O/100	6	80
6	0.1/EtOH/rt	2	20
7	0.1/EtOH/78	2	50
8	0.15/H_2_O/100	6.5	80
9	0.25/H_2_O/100	7	50
10	0.3/H_2_O/100	7	60

In order to affirm the crucial promotional influence of the bio-nanocomposite on the model reaction, the synthesis of the product **4o** was probed in the presence of each layer of the final nanostructure, as well as under catalyst-free conditions. The resultant data are shown in Table 4. The observations affirmed which the presence of each shell on the core influenced the reaction progress. This can be attributed to the combined catalytic effect of each constituent in the entire bio-nanocomposite that synergistically developed its catalytic potential. It must be mentioned that all the entries performed within 6 h.

**Table 4:** Investigation of the bio-nanocomposite efficacy for the preparation of **4n.**

Entry	Catalyst (0.1 g)/H_2_O (3 mL)/100 °C	Yield (%)
1	–	–
2	Co_0.2_Zn_0.6_Cu_0.2_Fe_2_O_4_	10
3	Co_0.2_Zn_0.6_Cu_0.2_Fe_2_O_4_-SiO_2_	20
4	[GuaH]^+^[Tar]^2‒^[GuaH]^+^ IL	20
5	Co_0.2_Zn_0.6_Cu_0.2_Fe_2_O_4_-SiO_2_@[GuaH]^+^[Tar]^2‒^[GuaH]^+^	80

As demonstrated in Table 5, various 5,10-dihydropyrido[2,3-d:6,5-*d'*]dipyrimidine-2,4,6,8(1H,3H,7H,9H)-tetraones were successfully achieved according to the optimized conditions. The products **4a-e** were obtained through the reaction of (+)-*D*-glucose (**1a**) with aniline and its derivatives (with electron-withdrawing and electron-donating substituents). The reaction of benzylamine (**3g**) with (+)-*D*-glucose (**1a**) produced **4f** successfully. Replacing this carbohydrate with (+)-*D*-galactose (**1b**) also yielded acceptable results (**4g-4h**). (+)-*D*-mannose (**1c**), as another aldohexose, reacted well with 4-methoxy aniline to produce the compound **4i**. Performing the reaction with (+)-*D*-fructose (**1d**), as a ketohexose derivative, yielded the appropriate polyhydroxy-substituted pyridine-dipyrimidine fused heterocycles with excellent yield (**4j**). (−)-*L*-arabinose (**1e**) and (+)-*D*-xylose (**1f**), as aldopentose sugars, provided the corresponding products with satisfactory results (**4k-4m**). The wide effectiveness of the procedure was affirmed through the utilization of lactose and maltose, as disaccharide candidates, to produce the numerous products **4n-4o** with good yields. The known products were characterized by comparing their obtained data with their genuine reports [52,53]. The spectral data of the novel derivatives (**4e, 4j, 4l**) are depicted in the experimental section.

**Table 5:**
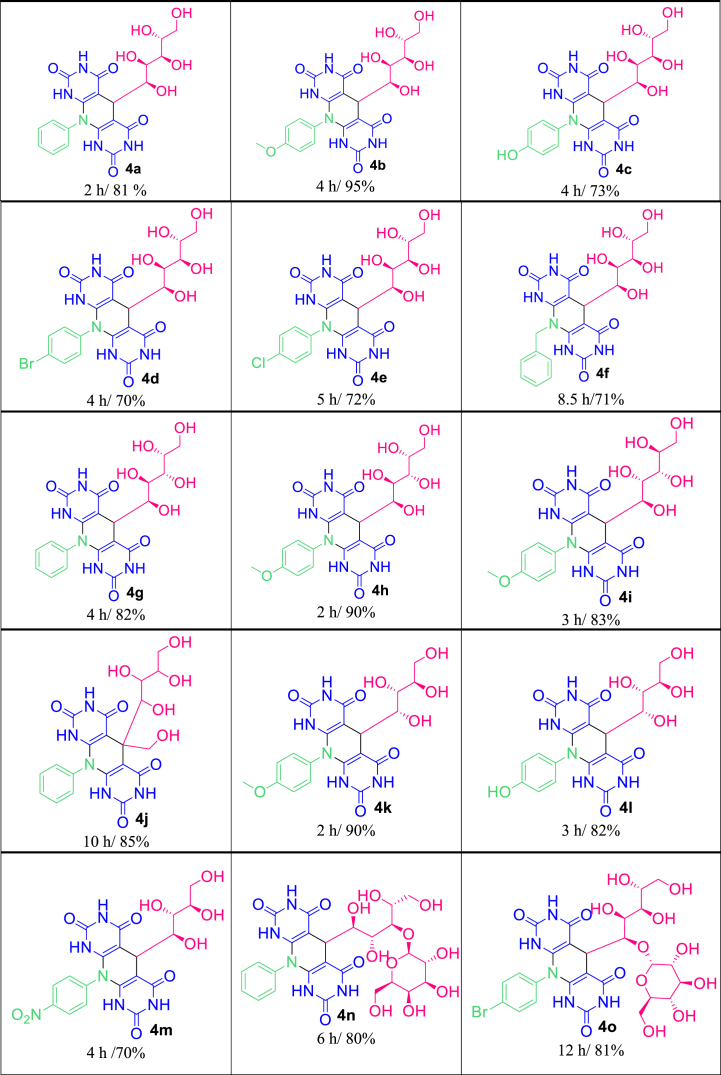
Synthesis of polyhydroxy pyrimidine-fused heterocycles (PPFHs) **4a-o** in the presence of CZCF-SiO_2_@[GuaH]^+^[Tar]^2‒^[GuaH]^+^ (0.1 g) in H_2_O (3 mL) at 100 °C.

**Fig. 14:**
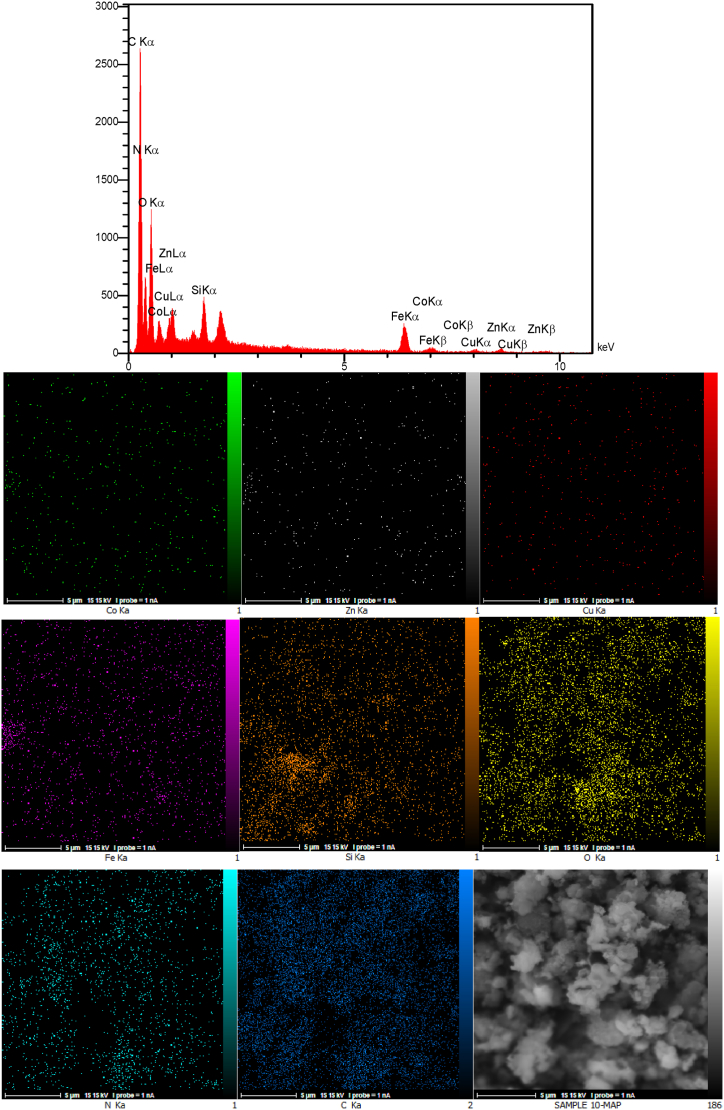
EDAX analysis and elemental mapping of the reused Co_0.2_Zn_0.6_Cu_0.2_Fe_2_O_4_-SiO_2_@[GuaH]^+^[Tar]^2‒^.

**Fig. 15:**
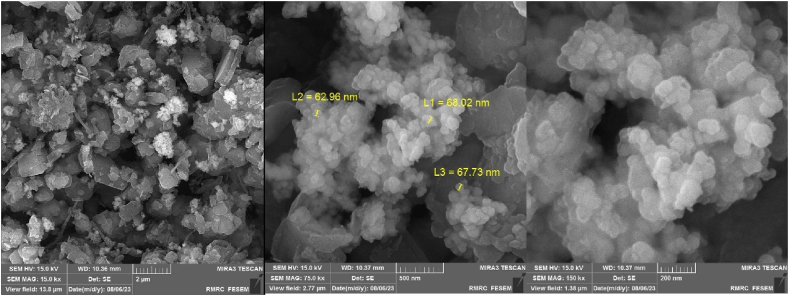
FESEM images of the recovered Co_0.2_Zn_0.6_Cu_0.2_Fe_2_O_4_-SiO_2_@[GuaH]^+^[Tar]^2‒^.

To elucidate the recyclability and reusability of the bio-nanocomposite, the model reaction was investigated with 2 runs. Product **4n** was achieved at 80 % and 78 % yield in the first and second runs, respectively. After the completion of each cycle, the bio-nanocatalyst was separated by an external magnet, washed with MeOH (2 × 5 mL), and finally air-dried. The recycled and reused catalyst was characterized via EDAX/mapping analysis (Fig. 14) and FESEM imaging (Fig. 15). The EDAX data affirmed that the recycled Co_0.2_Zn_0.6_Cu_0.2_Fe_2_O_4_-SiO_2_@[GuaH]^+^[Tar]^2‒^[GuaH]^+^ consisted of cobalt (0.16 wt%), zinc (0.94 wt%), copper (0.50 wt%), iron (3.44 wt%), oxygen (26.60 wt%), silicon (0.92 wt%), carbon (34.43 wt%), and nitrogen (33.02 wt%). Based on EDAX mapping analysis, the distribution of Co, Zn, Cu, Fe, O, Si, C, and N appeared homogeneous in the bio-nanostructure.

The FESEM imaging (at 2 μm, 500 nm and 200 nm magnification) also showed some agglomeration occurring in the bio-nanocomposite compared to the fresh nanostructure. The average diameter of the nanoparticles was 60–70 nm (Fig. 14).

In order to investigate the efficiency of our method, the preparation of 5-((1S,2R,3S,4R)-1,2,3,4,5-pentahydroxypentyl)-10-phenyl--5,10-dihydropyrido[2,3-*d*:6,5-*d'*]dipyrimidine-2,4,6,8(1*H*,3*H*,5*H*,7*H*)-tetraone (**4g**) (which obtained via the pseudo four-component reaction of *D*-galactose, barbituric acid, and aniline) was compared with previously reported procedures (Table 6).

**Table 6:** Comparison the methods for the preparation of **4g**.

Entry	Conditions	Time (h)	Yield (%)	References
1	*p*-TSA (0.1 g)/EtOH/50 °C	12	84	[52]
2	NCC (0.024 g)/EtOH/reflux	2	71	[53]
3	Co_0.2_Zn_0.6_Cu_0.2_Fe_2_O_4_-SiO_2_@[GuaH]^+^[Tar]^2‒^[GuaH]^+^ (0.1 g)/H_2_O/reflux	4	82	This work

## 3 Experimental

### 3.1 General

All chemicals were purchased from Aldrich, Merck, and Alfa-Aesar companies and utilized without any further purification. Melting points were obtained using an Electrothermal 9200 apparatus and reported uncorrected. FT-IR spectra were prepared using KBr disks on a Bruker Tensor 27 spectrometer. Magnetization properties were recorded by a VSM (LBKFB model) apparatus. Thermal gravimetric analysis (TGA) was evaluated by SDT Q600 V20.9 Build 20 instrument. The HRTEM data were recorded using an HR-TEM FEI TECNAI F20 apparatus. The XRD patterns were performed on a Philips apparatus employing Cu Kα1 radiation and a Co anode (λ = 1.78897 Å), 40 kV, and 40 mA. Elemental examinations were carried out utilizing a Philips-PW2404 XRF spectrometer. The centrifuge apparatus was a UNIVERSAL 320 (5000–10000 rpm) for the preparation procedure of the nanocatalyst. Morphology and size were assessed utilizing FESEM (VEGA\\TESCAN-LMU). The EDAX analysis was conducted using a TESCAN MIRA III machine. Mass spectra were achieved on 5973 Network Mass Selective Detector (Agilent Technology, HP). The ^1^H NMR and ^13^C NMR spectra were taken by Bruker drx spectrometer in the presence of DMSO-*d*_*6*_ as solvent. The zeta potential analysis was done by a SZ-100z Dynamic Light Scattering & Zeta potential analyzer from Horiba Jobin Jyovin company. The aqua suspension of the bio-nanocomposite at 25 °C with various pH = 2, 4, 6, 7, 8, 10, and 12 is used to draw the diagram.

### 3.2 Synthetic procedure for Co_0.2_Zn_0.6_Cu_0.2_Fe_2_O_4_-SiO_2_@[GuaH]^+^[Tar]^2‒^[GuaH]^+^ bio-nanocomposite

#### 3.2.1 Synthesis of Co_0.2_Zn_0.6_Cu_0.2_Fe_2_O_4_

The Co_0.2_Zn_0.6_Cu_0.2_Fe_2_O_4_ prepared through a modified co-precipitation method [49,54]. Typically, deionized water was added to the mixture of CoCl_2_·6H_2_O, anhydrous ZnCl_2_, Cu(NO_3_)_2_·3H_2_O, and anhydrous FeCl_3_ in a stoichiometric ratio of 0.2:0.6:0.2:2, to obtain a totally homogeneous solution. Then, a few drops of concentrated hydrochloric acid were added to achieve a clear solution, which followed by heating at 80 °C for 3 h. After that, a NaOH (0.4 M) solution added to the stirring mixture to reach the pH = 10. After precipitation, the mixture was stirred magnetically for 4 h at 80 °C. To gain a neutral pH condition, the slurry washed several times with deionized water, and subsequently oven-dried at 100 °C for 12 h. The mixed spinel CZC- ferrite obtained by annealing the solid at 800 °C for 3 h.

#### 3.2.2 Silica embedding on CZC ferrite

In a typical manner, Co_0.2_Zn_0.6_Cu_0.2_Fe_2_O_4_ nanoparticles (1 g) were dispersed in a mixture of 25 wt% ammonia (2 mL), deionized water (20 mL), and absolute ethanol (60 mL). The suspension sonicated in a bath for 30 min. Subsequently, a mixture of tetraethyl orthosilicate (TEOS, 0.5 mL) in absolute ethanol (1 mL) was added dropwise under vigorous stirring at room temperature and the mixture was stirred for 20 h at room temperature. Finally, the solid was separated by an external magnet, washed with absolute ethanol (3 × 5 mL), and dried at 70 ᵒC for 5 h to obtain Co_0.2_Zn_0.6_Cu_0.2_Fe_2_O_4_-SiO_2_ [55].

#### 3.2.3 Preparation of [GuaH]^+^[Tar]^2‒^[GuaH]^+^ ionic liquid

A mixture of guanine (2 mmol) and *L*-tartaric acid (1 mmol) in deionized water (5 mL) was refluxed for 10 h. The solid was separated via centrifuging and washing with absolute ethanol (3 × 5 mL). After air-drying the residue for 2 h, the final [GuaH]^+^[Tar]^2‒^[GuaH]^+^ IL was obtained by heating at 70 °C for 5 h as white powder. Mp = 330 °C. ^1^H NMR (500 MHz, DMSO-*d*_*6*_): 4.29 (bs, 2H, OH), 5.44 (d, *J* = 7.55 Hz, 2H, -CH), 7.36–7.39 (m, 4h, -Ar + NH), 10.79 (s, 4H, NH_2_), 10.99 (s, 2H, NH). ^13^C NMR (125.5 MHz, DMSO-*d*_*6*_): 72.61, 100.71, 142.69, 157.03, 164.88. MS (ESI) *m*/*z* 452 [M]^₊^.

### 3.3 Synthesis of Co_0.2_Zn_0.6_Cu_0.2_Fe_2_O_4_-SiO_2_@[GuaH]^+^[Tar]^2‒^[GuaH]^+^

A mixture of Co_0.2_Zn_0.6_Cu_0.2_Fe_2_O_4_-SiO_2_ nanostructure (1 g) and IL (1 g) in EtOAc (10 mL) and HCl (1 M, 0.5 mL) was stirred under reflux conditions for 5 h. Then, the solid was separated using an external magnet and washed with EtOAc (3 × 10 mL). After air-drying (5 h), the solid residue was heated in an oven at 50 °C for 5 h to obtain the final bio-nanocomposite.

### 3.4 General procedure for the synthesis of polyhydroxy-substituted pyridine-dipyrimidine fused heterocyclic compounds

To a solution of carbohydrates (**1a-h**, 1 mmol), barbituric acid (**2**, 2 mmol), and amines (**3a-g**, 1 mmol), in water (3 mL), the nano Co_0.2_Zn_0.6_Cu_0.2_Fe_2_O_4_-SiO_2_@[GuaH]^+^[Tar]^2‒^[GuaH]^+^ (0.1 g) was added. The mixture was refluxed for the appropriate time and monitored by (MeOH/EtOAc eluent, 1:2). After completion, the nanocatalyst was separated utilizing an external magnet and washed with EtOH (2× 5 mL). The elimination of the solvent under reduced pressure afforded almost pure compounds (**4a-o**) which were further purified by recrystallization from EtOH.

10-(4-Chlorophenyl)-5-((1S,2R,3R,4R)-1,2,3,4,5-pentahydroxypentyl)-5,10-dihydropyrido[2,3-*d*:6,5-*d*']dipyrimidine-2,4,6,8(1*H*,3*H*,7*H*,9*H*)-tetraone (4e)

Brown powder; mp = 176 °C; IR (KBr, cm^−1^): 3419, 3214, 3036, 2893, 2663, 1716, 1637, 1511, 1490, 1373, 1203, 1180, 1126, 1097, 1035, 1010, 816, 683, 568, 495. ^1^H NMR (300 MHz, DMSO-*d*_*6*_) *δ* (ppm): 1.053–1.17 (m, 3H, -CH), 3.07–3.56 (m, 4H, -CH), 3.62–3.97 (m, 5H, -OH), 7.14–7.59 (m, 4H, Ar), 10.68 (brs, 1H, NH), 11.15–11.3 (brs, 1H, NH), 11.3–11.4 (brs, 2H, NH). ^13^C NMR (75 MHz, DMSO-*d*_*6*_) *δ* ppm: 15.52, 21.26, 32.13, 61.48, 64.25, 67.07, 69.92, 70.87, 72.40, 79.65, 84.68, 92.15, 98.77, 125.32, 125.95, 128.71, 130.22, 131.79, 132.71, 138.63, 145.42, 150.29, 157.57, 164.98. MS (ESI): *m*/*z* 509.6 [M]^+^.

5-(Hydroxymethyl)-10-phenyl-5-(1,2,3,4-tetrahydroxybutyl)-9,10-dihydropyrido[2,3-*d*:6,5-*d*']dipyrimidine-2,4,6,8(1*H*,3*H*,5*H*,7*H*)-tetraone (4j)

Brown powder; m p = 116 °C; IR (KBr, cm^−1^): 3422, 2925, 2646, 1702, 1649, 1540, 1500, 1455, 1420, 1396, 1196, 1122, 1034, 1009, 813, 749, 681, 565, 474, 419. ^1^H NMR (300 MHz, DMSO-*d*_*6*_) *δ* (ppm): 3.22–3.31 (m, 3H, -CH), 3.32–3.83 (m, 4H, -CH), 4.1 (brs, 1H, -OH), 4.21 (brs, 1H, -OH), 4.52 (brs, 1H, -OH), 4.7 (s, 2H, -OH), 7.15–7.17 (m, 2H, -Ar), 7.39–7.48 (m, 2H, -Ar), 7.51 (m, 1H, -Ar), 7.54 (brs, 2H, NH), 11.28 (brs, 2H, NH). ^13^C NMR (75 MHz, DMSO-*d*_*6*_) *δ* ppm: 21.27, 40.14, 40.41, 40.69, 61.68, 62.29, 63.80, 69.35, 78.48, 83.84, 96.01, 101.64, 123.77, 125.96, 128.82, 128.90, 129.17, 130.30, 131.87, 138.95, 144.95. MS (ESI): *m*/*z* 475 [M]^+^.

10-(4-Hydroxyphenyl)-5-((1R,2S,3R)-1,2,3,4-tetrahydroxybutyl)-5,10-dihydropyrido[2,3-*d*:6,5–-*d*']dipyrimidine-2,4,6,8(1*H*,3*H*,7*H*,9*H*)-tetraone (4l)

Brown powder; mp = 215–216 °C; IR (KBr, cm^−1^): 3574, 3470, 3322, 2975, 2664, 2623, 1709, 1641, 1553, 1522, 1457, 1366, 1222, 1158, 1125, 1032, 1007, 835, 814, 686, 567, 510, 464. ^1^H NMR (300 MHz, DMSO-*d*_*6*_) *δ* (ppm): 3.33 (m, 1H, -CH), 3.36–3.68 (m, 2H, -CH), 3.72–3.76 (brs, 3H, -CH), 4.09–4.27 (brs, 2H, -OH), 4.34–4.38 (brs, 2H, -OH), 6.86–6.89 (d, *J* = 8.4 Hz, 1H, -Ar), 7.15–7.21 (m, 1H, -Ar), 7.54–7.57 (d, *J* = 7.55 Hz, 1H, -Ar), 9.81 (brs, 1H, -OH), 10.85 (s, 1H, -NH), 11.22 (brs, 2H, -NH), 11.40 (s, 1H, -NH). ^13^C NMR (75 MHz, DMSO-*d*_*6*_) *δ* ppm: 21.26, 40.11, 40.39, 40.66, 56.57, 116.60, 122.62, 124.81, 125.95, 128.83, 139.00, 144.91, 157.72. MS (ESI): *m*/*z* 461 [M]^+^.

### 3.5 Computational details

Considering the wide application of density functional theory (DFT) calculations in ionic liquids (ILs) [56,57] and the hydrogen bond investigations [58,59], all calculations were performed at the B3LYP/6–311++G∗∗ computational level using the Gaussian 03 suite of programs [60]. All molecular structures were fully optimized in the water (ε = 78.39) without any symmetry constraints, using the self-consistent reaction field (SCRF) and polarizable continuum model (PCM) [61]. The frequency calculations of ionic liquid constituents were calculated in the water solvent. The Mercury [62] and Avogadro [63] programs were employed to visualize the molecular graphics and molecular electrostatic potential (MESP) surfaces. The interaction energies ($E_{\mathrm{int}})$ of intermolecular hydrogen bonds (IHBs) [64] were evaluated by Eq. (1).

(1) $$E_{\mathrm{int}}=E_{Tar-Gu}-\left(E_{Tar}+E_{Gu}\right)$$

where $E_{Tar-Gu}$, $E_{Tar}$, and $E_{Gu}$ are the total electronic energies of the fully optimized structures of the Tartaric acid-Guanine (Tar-Gu) complex and isolated molecules of Tar and Gu in the solvent. The zero-point corrected (ZP Corr.) Energies were calculated by considering the sum of the electronic and zero-point energies of the optimized structures. The interaction energies of ILs [65] were defined as follows:

(2) $$E_{\mathrm{int}}=E_{IL}-\left(E_{cation}+E_{anion}\right)$$

The binding thermodynamic parameters at 298.15 K and 1.0 atm pressure, including the standard Gibbs free energy change (ΔG°), the standard enthalpy change (ΔH°), and the standard entropy change (ΔS°) were calculated according to Eqs. (1), (2)), where the electronic energies were replaced with the Gibbs free energy, enthalpy, and entropy of constituents in the solvent. The solvation energy (SE) was estimated by the difference in Gibbs free energy associated with transforming the optimized structure of the molecule from the gas phase to the liquid phase.

## 4 Conclusions

In summary, a new magnetized multi-layered bio-nanocomposite (Co_0.2_Zn_0.6_Cu_0.2_Fe_2_O_4_-SiO_2_@[GuaH]^+^[Tar]^2‒^[GuaH]^+^) was prepared successfully through a multi-step simple synthetic process and subsequently characterized using FT-IR, EDAX, FESEM, XRF, XRD, TGA/DTG, VSM, and HRTEM techniques. The catalytic assessment of the inorganic-bioorganic nanostructure was examined for the generation of polyhydroxy-substituted pyridine-dipyrimidine fused heterocyclic compounds via the pseudo four-component condensation reactions in water under reflux conditions. The recoverability and reusability of the bio-nanostructure were checked up to 3 runs without any considerable loss of efficiency. Additionally, the FESEM and EDAX analysis of the recycled nanocatalyst indicated the stability of its structure. Furthermore, the detailed computational studies affirmed the structure of the novel bio-IL in the nanocomposite.

## CRediT authorship contribution statement

**Zahra Khademi:** Writing – original draft, Methodology, Investigation. **Kobra Nikoofar:** Writing – review & editing, Writing – original draft, Supervision, Formal analysis. **Mansoureh Zahedi-Tabrizi:** Writing – original draft, Software, Formal analysis.

## Data availability statement

Data included in article/supp. material/referenced in article.

## Declaration of competing interest

The authors declare that they have no known competing financial interests or personal relationships that could have appeared to influence the work reported in this paper.
