# Supplementary material for: Trimetallic ferrite functionalized by guaninium tartrate ionic liquid (Co0.2Zn0.6Cu0.2Fe2O4-SiO2@[GuaH]+[Tar]2‒[GuaH]+) as a novel inorganic-bioorganic nanostructure to promote aqua-mediated synthesis of polyhydroxy-substituted pyridine-dipyrimidine fused heterocycles
Source: Heliyon. 2025 Feb 4;11(3):e42462. doi: 10.1016/j.heliyon.2025.e42462 (PMC11850137; doi:10.1016/j.heliyon.2025.e42462)
Supplement: Multimedia component 1 [file mmc1.docx]

**Trimetallic ferrite functionalized by guaninium tartrate ionic liquid (**[**Co_0.2_Zn_0.6_Cu_0.2_Fe_2_O_4_-SiO_2_@[GuaH]^+^[Tar]^2‒^**](mailto:Co0.2Zn0.6Cu0.2Fe2O4-SiO2@Gua+Tar-)**[GuaH]^+^) as a novel inorganic-bioorganic nanostructure to promote aqua-mediated synthesis of polyhydroxy-substituted pyridine-dipyrimidine fused heterocycles**

Zahra Khademi^a^, Kobra Nikoofar^a,^*, Mansoureh Zahedi-Tabrizi^b^

*^a^ Department of Organic Chemistry, Faculty of Chemistry, Alzahra University, Tehran, Iran*

*^b^ Department of Physical Chemistry & Nanochemistry, Faculty of Chemistry, Alzahra University, Tehran, Iran*

*Corresponding author email: k.nikoofar@alzahra.ac.ir; [kobranikoofar@yahoo.com](mailto:kobranikoofar@yahoo.com)


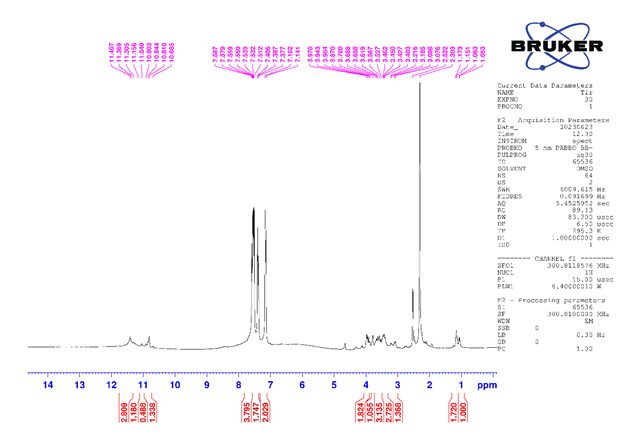


**SI-1.** ^1^H NMR (300 MHz, DMSO-*d_6_*) of **4e**


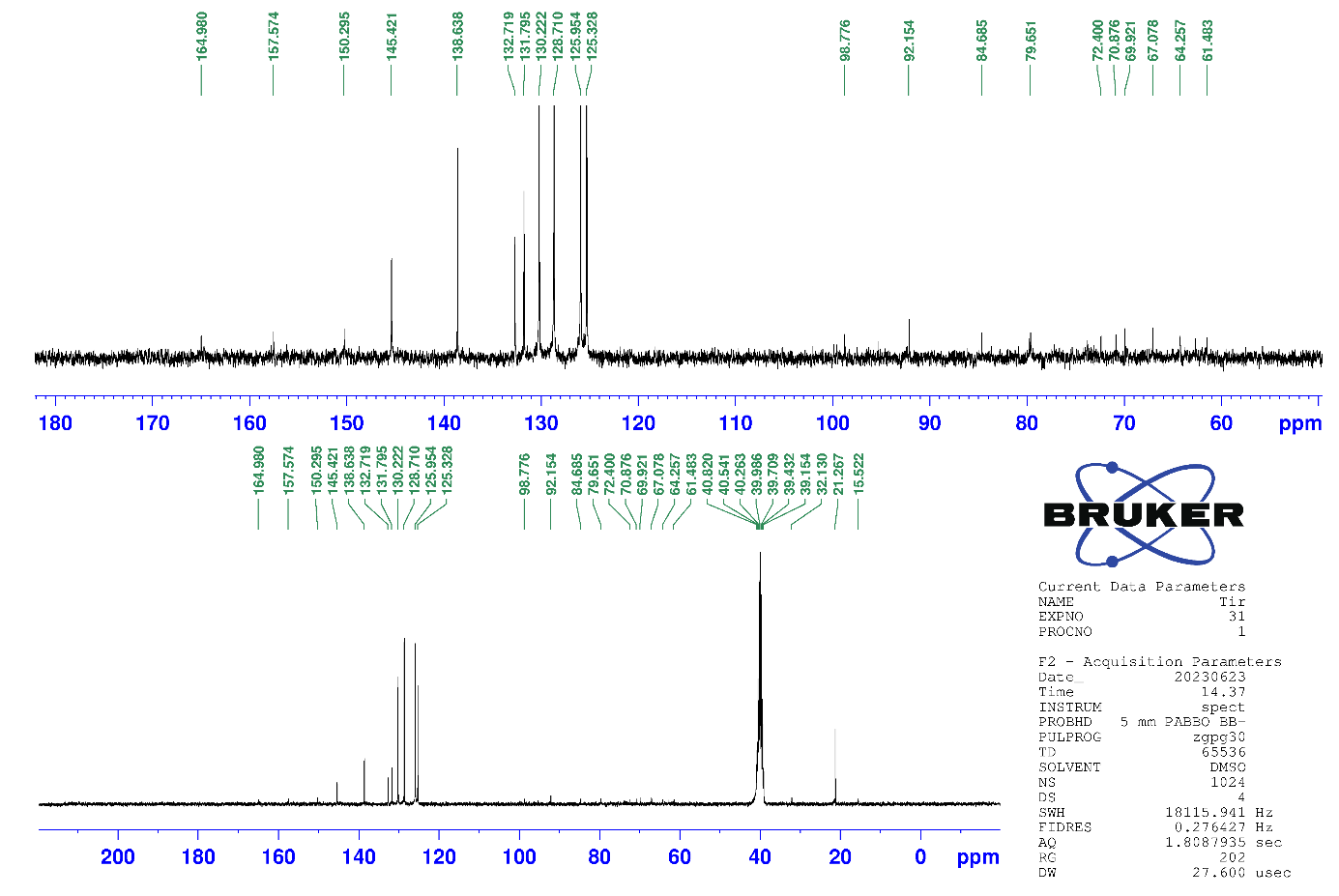


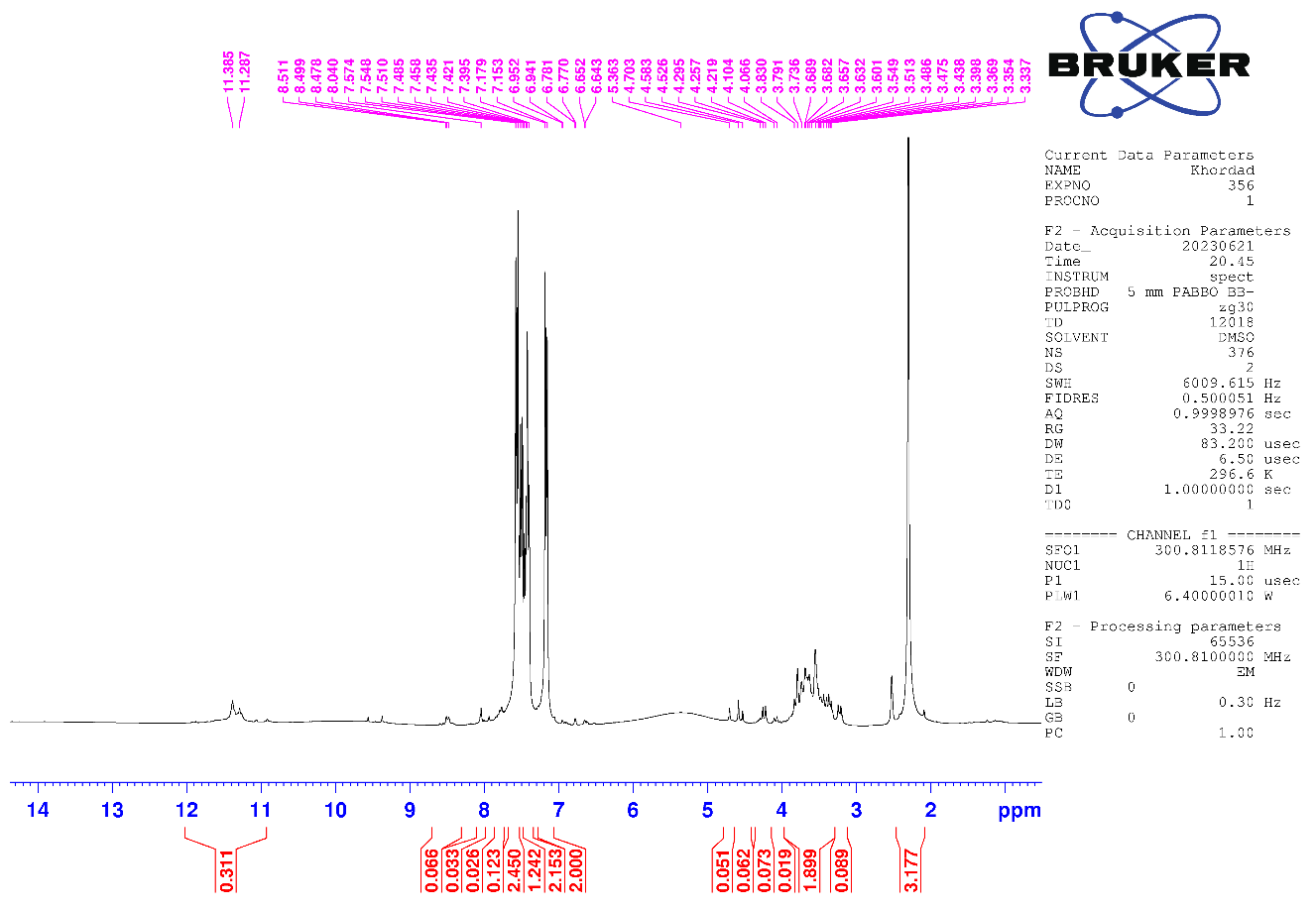
**SI-2.** ^13^C-NMR (75 MHz, DMSO-*d_6_*) of **4e**

**SI-3.** ^1^H NMR (300 MHz, DMSO-*d_6_*) of **4j**


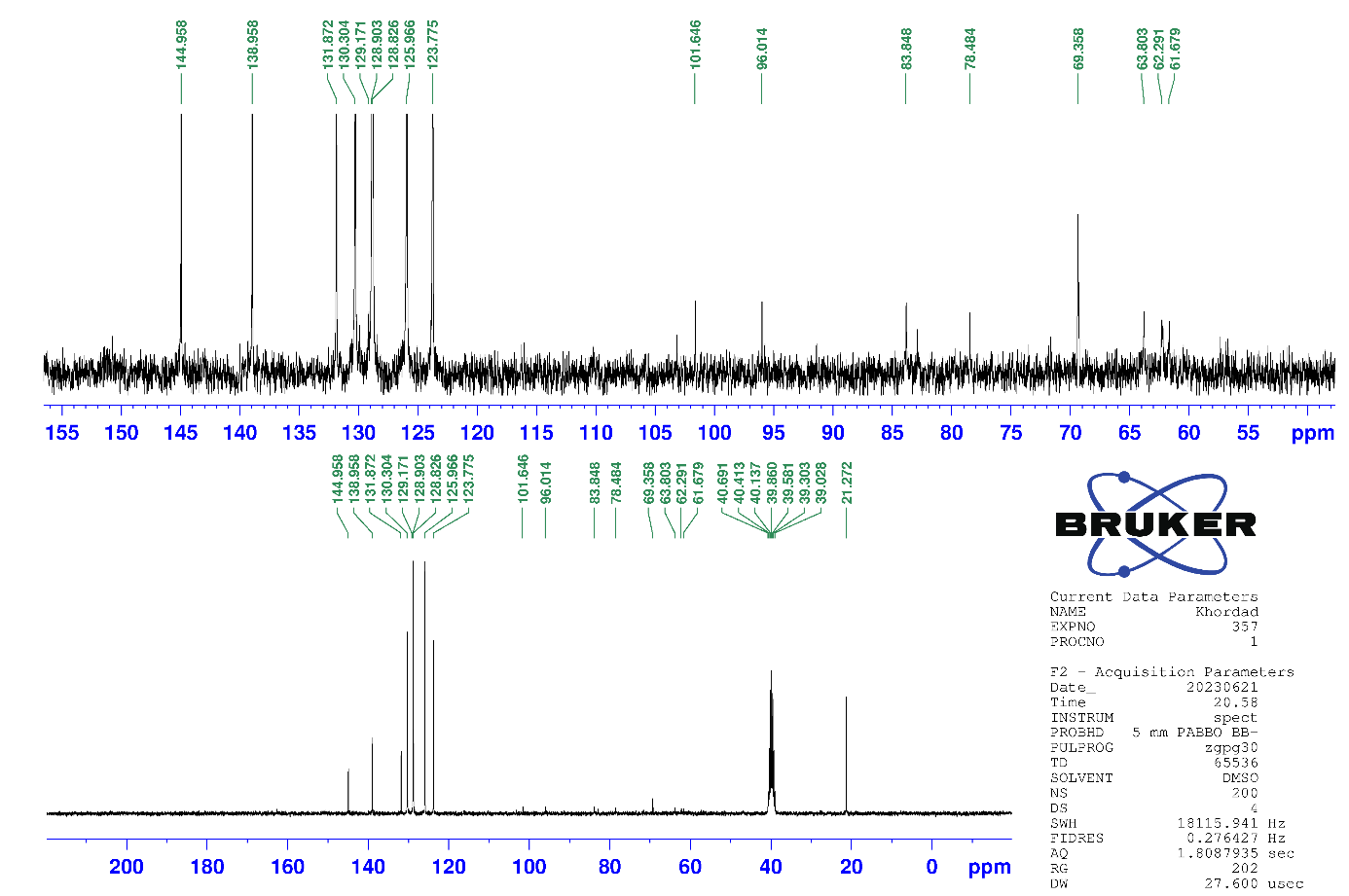


**SI-4.** ^13^C-NMR (75 MHz, DMSO-*d_6_*) of **4j**


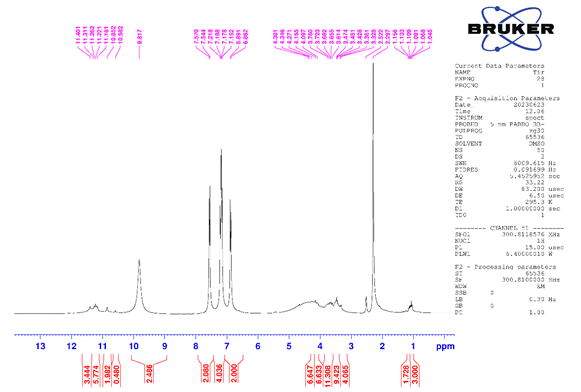


**SI-5.** ^1^H NMR (300 MHz, DMSO-*d_6_*) of **4l**

**
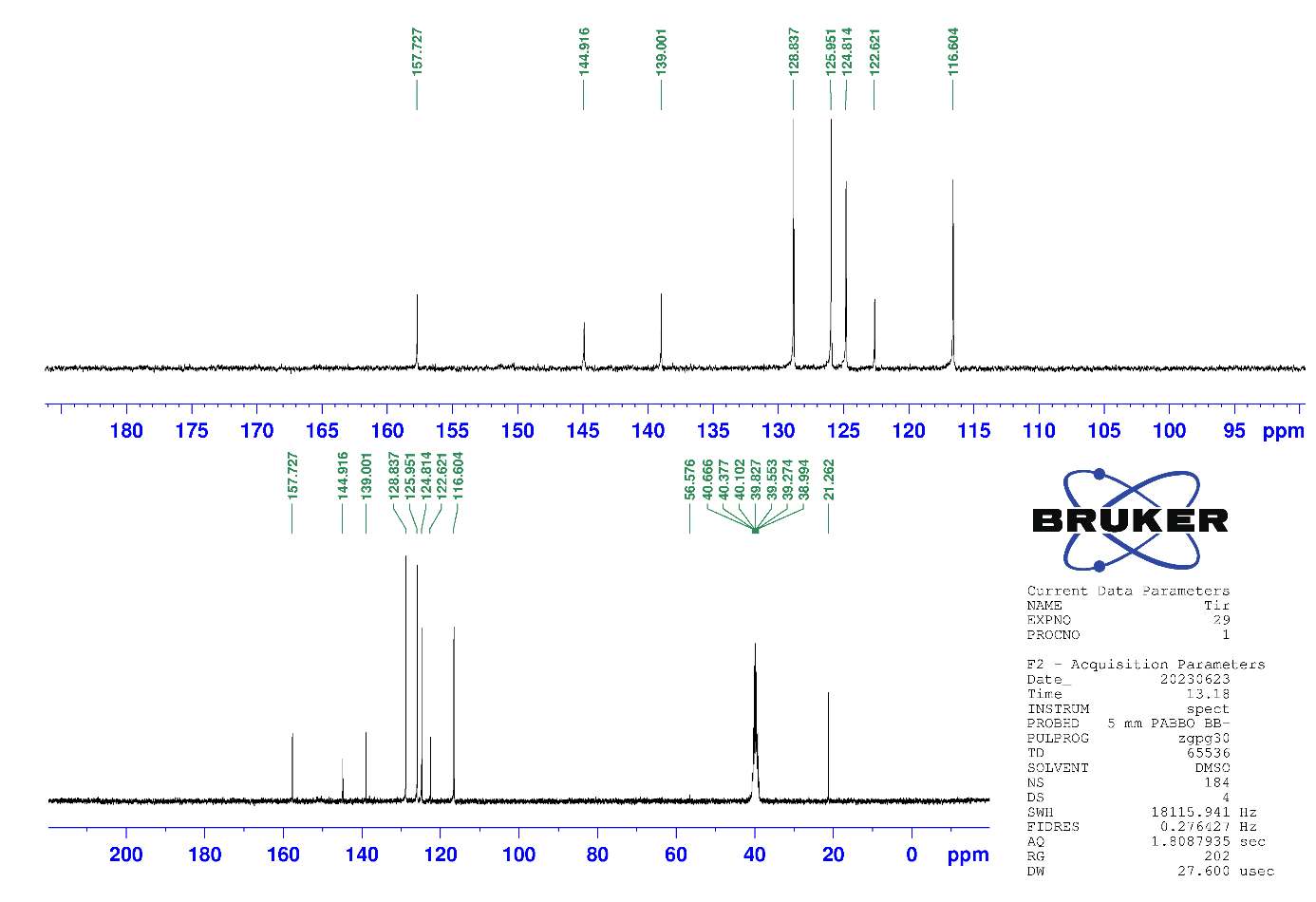
**

**SI-6.** ^13^C-NMR (75 MHz, DMSO-*d_6_*) of **4l**
